# A KDEL Retrieval System for ER-Golgi Transport of Japanese Encephalitis Viral Particles

**DOI:** 10.3390/v8020044

**Published:** 2016-02-05

**Authors:** Robert YL Wang, Yu-Jen Wu, Han-Shan Chen, Chih-Jung Chen

**Affiliations:** 1 Department of Biomedical Sciences and Molecular Medicine Research Center, Chang Gung University, Taoyuan 33302, Taiwan; 2 Graduate Institute of Biomedical Sciences, College of Medicine, Chang Gung University, Taoyuan 33302, Taiwan; h318joe@yahoo.com.tw; 3 Department of Food Science and Nutrition, Meiho University, Pingtung 91202, Taiwan; wyr924@ms24.hinet.net; 4 Division of Pediatric Infectious Diseases, Department of Pediatrics, Chang Gung Memorial and Children’s Hospital, Linkuo 33375, Taiwan

**Keywords:** Japanese encephalitis virus, GRP78, KDEL receptor, ER-Golgi retrieval, viral particles

## Abstract

Evidence has emerged that RNA viruses utilize the host secretory pathway for processing and trafficking mature viral particles and for exiting the infected cells. Upon completing the complex assembly process, the viral particles take advantage of the cellular secretory trafficking machinery for their intracellular trafficking toward the Golgi organelle and budding or export of virions. In this study, we showed that Japanese encephalitis virus (JEV)-induced extracellular GRP78 contains no KDEL motif using an anti-KDEL-specific antibody. Overexpression of the KDEL-truncated GRP78 in the GPR78 knocked down cells significantly reduced JEV infectivity, suggesting that the KDEL motif is required for GRP78 function in the release of JE viral particles. In addition, we demonstrated the KDELR protein, an ER-Golgi retrieval system component, is associated with viral envelope proteins and is engaged in the subcellular localization of viral particles in Golgi. More importantly, accumulation of intracellular virions was observed in the KDELR knocked down cells, indicating that the KDELR protein mediated the intracellular trafficking of JE viral particles. Altogether, we demonstrated that intracellular trafficking of JE assembled viral particles was mediated by the host ER-Golgi retrieval system prior to exit by the secretory pathway.

## 1. Introduction

Japanese encephalitis virus (JEV) is a mosquito-borne flavivirus that causes human epidemic encephalitis; it is mostly found in Asian countries, including Japan, China, Taiwan, South Korea and India; and it leads to a 10% to 40% fatality rate [1]. Other medically prominent pathogens, such as dengue virus (DENV), yellow fever virus, West Nile virus, Murray Valley encephalitis virus and tick-borne encephalitis virus, belong to the same family of *Flaviviridae*. The JEV genome is a single-stranded, positive-sense RNA comprising 10,976 nucleotides (nts) and containing a 5′ capped structure, but no polyA tail at its 3′ end. The JE viral RNA genome is a large open reading frame flanked by 5′ and 3′ untranslated regions and encoding a large polyprotein that is processed into three structural proteins, the capsid (C), premembrane (prM) and envelope (E) proteins, and seven nonstructural (NS) proteins, the NS1, NS2A, NS2B, NS3, NS4A, NS4B and NS5 proteins.

The transport and release of newly-assembled JE viral particles is a complex and dynamic process that involves hijacking host membrane transport systems, including the endoplasmic reticulum (ER) to the Golgi classic secretory pathway, by using viral structural proteins [2]. After viral envelope protein assembly and genome packaging at the site of an ER-derived membrane, viral particles exit the ER and traffic to a site of secondary envelopment considered to be trans-Golgi or endosomal membranes. The JE viral particles transport vesicles and then traffic to the plasma membrane, where the viral particles are released from the infected cell by exocytosis. Little is known about the intracellular trafficking of JEV particles from the ER to the plasma membrane. Similar to other flaviviruses, JE viral replication and the assembly of viral particles are localized in ER-derived luminal membranes and subsequently sorted to the lumen of other organelles or are secreted from the cell [3]. Similarly, initial envelopment by some viral envelope proteins is associated with the rough ER membrane during their synthesis; some remain there, but many eventually become localized at the Golgi complex, lysosomes or endosome [4,5].

Cells infected with viruses generally undergo a remodeling of their intracellular membrane for viral replication and become sites of viral particle envelopment [6]. Subsequently, newly-enveloped viral particles move alone along the cellular secretory pathway, which includes a number of separate compartments. The KDEL receptor (KDELR) protein is a transmembrane protein identified as a component involved in the ER-retrieval system, because the KDELR protein was shown to be transported between the ER and Golgi organelle. Chaperones, such as glucose regulation protein 78 (GRP78), GRP94 and protein disulfide isomerase (PDI), transport cellular cargos through the ER-retrieval system, with the C-terminal tetrapeptide KDEL motif shared by several ER chaperones. When a cell is infected, a virus may benefit from this transport pathway to deliver enveloped viral particles for its trafficking route and exit to the cell surface. In vaccinia virus-infected HeLa cells, two host proteins, coatomer and KDELR, participated in vaccinia viral formation to form novel transport carriers for delivering host cellular membranes to early viral forms [7]. Therefore, as a cellular homeostatic system for the retrograde transport of proteins, the KDELR protein can be hijacked by a virus that must be transported along the secretory pathway to the Golgi complex, subsequently exiting the cell surface [8].

We previously showed that JEV coopted GRP78 in the assembly of virions, as well as for subsequent viral infectivity [9]. In addition, the association of GRP78 with virions was observed in the sucrose-density-gradient purification and by dual-immunofluorescent staining *in vivo*. Because GRP78 is one of the ER luminal proteins and serves the cellular cargo in the ER-retrieval system, the binding of GRP78 with JE viral envelope proteins may be involved in sorting newly-assembled JEV particles en route from the ER to the Golgi and in their subsequent exits from the cell. In this study, we continued exploring the virus-induced secretion of extracellular ER luminal proteins and the influence of the C-terminus KDEL motif of GRP78 on virus infectivity in JEV-infected BHK-21 cells. In addition, the KDELR protein was demonstrated to be involved in the ER trafficking of JEV particles, targeting the Golgi organelle prior to being released from the cells. A significant decrease of extracellular viral RNA and viral infectivity was detected in siKDELR-treated cells. More importantly, we observed an accumulation of intracellular-assembled JEV particles in close association with the ER replication complex in the knocked down expression of KDELR protein in JEV-infected cells. These results suggested that the KDELR protein, an ER-Golgi retrieval system component, mediated the intracellular trafficking of the JEV particles.

## 2. Materials and Methods

### 2.1. Virus and Cell Culture

The cultivation of BHK-21 cells and amplification of JE virus stock are described in previous studies [9,10]. The JE virus used in this study was the T1P1 strain isolated from field-caught *Armigeres subalbatus* [11] (provided by Wei-June Chen, Chang Gung University, Taiwan).

### 2.2. Viral Plaque Assay

The plaque-forming assay is described in our previous study [10]. In brief, the BHK-21 cells were seeded in 6-well plates at 4 × 10^5^ cells per well, followed by overnight incubation in RPMI 1640 medium containing 2% FBS to form a monolayer. Before infection, serial 10-fold dilutions of the supernatant of JEV-infected medium were prepared in serum-free RPMI medium, followed by incubation of the JEV-infected BHK-21 cells with serum-free RPMI 1640 medium for 1 h, and then, 0.5 mL of 10-fold dilutions and 0.5 mL of serum-free RPMI 1640 medium were added per BHK-21 monolayer for 1.5 h. The 6-well tissue culture (TC) plates were incubated at room temperature for 30 min to allow the 0.3% agarose overlay to solidify, followed by incubation at 37 °C for 3 days. The cells were then fixed with formaldehyde and stained with crystal violet stain solution for 2 min. Finally, plaque-forming units (pfu/mL) were calculated using a virus titer formula, where the virus titer equaled the number of plaques × (1 mL/0.5 mL) × the dilution factor.

### 2.3. Immunoprecipitation, Western Blotting and Antisera

The mock- and JEV-infected cell lysates were collected and precleared using protein A agarose (Santa Cruz Biotechnology, Dallas, TX, USA) for background removal. The lysates were centrifuged at 1000× *g* for 5 min, and the supernatants were collected. Protein A agarose was blocked with 3% BSA for 30 min before binding the viral E- or KDELR-specific antibodies for 2 h at 4 °C. The supernatants were added to the protein A agarose beads. The mixture was incubated at 4 °C for 2 h, and the protein A agarose beads were washed 3 times with PBS. Regarding the Western blotting assay, the collecting and separating of the protein samples is described in a previous study [9]. The following antibodies were primarily used: mouse anti-JEV NS1/E/prM (1:3000 dilution) [9,10] (YaoHong Biotechnology, Inc., New Taipei City, Taiwan), mouse anti-KDELR1 (Novus Biologicals, Littleton, CO, USA), rabbit anti-KDELR (GeneTex, Irvine, CA, USA), rat anti-GPR94 (NeoMarkers, Fremont, CA, USA), rabbit anti-PDI (Santa Cruz Biotechnology) and rabbit anti-β-actin antiserum (Sigma, St. Louis, MO, USA).

### 2.4. Immunofluorescent Staining

For immunofluorescent staining, cells were cultured on glass coverslips, rinsed with PBS twice, fixed with 4% paraformaldehyde in PBS for 30 min at room temperature and then permeabilized with 0.1% (*v/v*) Triton X-100 in PBS for 30 min and incubated in 2% blocking buffer (Roche, Indianapolis, IN, USA) for 1 h. The primary antibodies used in our study are described in the “Immunoprecipitation, Western Blotting and Antisera” section. Rhodamine- or fluorescein-isothiocyanate-conjugated secondary antibodies were used. Images were acquired using a Zeiss confocal microscope and were processed using Adobe Photoshop software (Adobe systems incorporate, San Jose, CA, USA).

### 2.5. Sucrose Density Gradient Analysis

The cell lysates of JEV-infected BHK-21 cells (2 day post-infection at an Multiplicity of Infection (MOI) of 1) were centrifuged and concentrated prior to sucrose density gradient analysis. After a freeze-and-thaw treatment, the cell lysates were layered onto a 20% to 60% sucrose linear gradient in HEPES buffer (20 mM HEPES, 0.5 mM EDTA, 50 mM KCl) and centrifuged at 40,000 rpm for 17 h at 4 °C. Ten fractions (1 mL/fraction) were harvested from the top of the sucrose gradients.

### 2.6. shGRP78 Knocked down Stable Cells and siRNA Knocking down of KDELR Proteins

To generate the stable knockdown of GRP78 gene in the BHK-21 cells, we employed the BLOCK-iT Lentiviral U6 (ThermoFisher Scientific Inc., Waltham, MA, USA) plasmids with the designed targeted sequence of 5′GATAGATGTTAATGGTATT3′. After infection with the packaged lentiviral vector, the cells were grown with Zeocin selection pressure. The stable cells were picked and validated with the expression level of GRP78 protein by Western blotting. The KDELR1 siRNA 5′GUUCAAAGCUACUUACGAU3′ were synthesized by Sigma. A scrambled control siRNA was designed and synthesized by Invitrogen (Grand Island, NY, USA) (medium GC of StealthTM RNAi negative control duplex). The siRNA was transfected into cells using RNAiMAX lipofectamine (Invitrogen) in Opti-MEM reduced serum medium (Invitrogen). The siRNA was incubated with RNAiMAX for 30 min at room temperature prior to transfection, and the targeted protein expression levels were silenced during a 2-day incubation.

### 2.7. Construction of the GRP78 with C-Terminal Flag-Tagged Expression Plasmids

All primer sequences are written in the 5′ to 3′ orientation. To generate a full-length GFP-GRP78 plasmid, we amplified a full-length GRP78 by using RT-PCR to isolate the RNA of BHK-21 cells and used the forward primer, GCGCCTCGAGATGATGAAGTTCACTGTGGTG, and reverse primer, GCGCGGATCCCTACAACTCATCTTTTCTGATG. The PCR product was then treated with the restriction enzymes *Xho*I and *BamHI* and ligated into a pCMV-3Flag-8 vector previously digested with *Xho*I and *BamHI*. To generate the GPR78 without a KDEL motif, PCR was performed using the forward primer as described and the reverse primer GCGCGGATCCTTCTGATGTATCCTCTTCACC, followed by ligation into the pCMV-3Flag-8 vector.

### 2.8. Transmission Electron Microscopy

Cell monolayers were fixed with a primary fixative consisting of 1% glutaraldehyde (Agar Scientific, Stansted, UK) at 4 °C over 3 days. After a primary fixation, the cell monolayers were washed and scraped off before being postfixed with 1% osmium tetroxide (Ted Pella, Redding, CA, USA) for 2 h. A few grains of potassium ferrocyanide were added to enhance the contrast of the membranous structure within the cells. After 2 h, the cell pellets were washed and dehydrated with progressively increased concentrations of ethanol (25%, 50%, 75%, 95% and 100%). The dehydration step was enhanced by another 2 rounds of absolute acetone treatment for 10 min each. Dehydrated cell pellets were then infiltrated by adding increasing concentrations of araldite 502 (Ted Pella) to acetone at increasing temperatures before being embedded in fresh araldite for 24 h at 60 °C. The embedded samples were then trimmed with an ultramicrotome (Reichert-Jung, Depew, NY, USA) to approximately 50 to 70 nm. Cut sections were then placed onto a 200-mesh copper grid before being stained with 2% uranyl acetate and postfixed with lead citrate. Stained sections were viewed using the Philips EM 208 transmission electron microscope (Philips, Eindhoven, The Netherlands) and captured digitally with a dual-view digital camera (Gatan Inc., Werrendale, CA, USA).

## 3. Results

### 3.1. JEV Infection Activates the Expression and Secretion of ER Luminal Proteins

**Figure 1:**
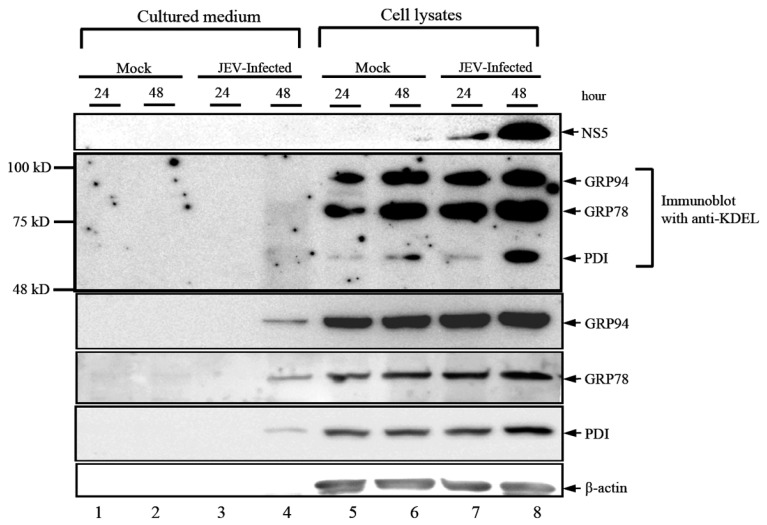
Detection of extracellular ER lumen proteins in the secreted medium from JEV-infected BHK-21 cells. BHK-21 cells were mock-infected or infected with Japanese encephalitis virus (JEV) at the MOI of 0.1, and both of the cultured medium (Lanes 1 to 4) and cell lysates (Lanes 5 to 8) at 24 h or 48 h (as shown on the lanes) were collected and determined for Western immunoblotting using antibodies specific for viral structural E protein, KDEL, GRP94, GRP78, PDI and b-actin, as indicated at the right. The cytoplasmic forms of the ER lumen proteins, GRP94, GRP78 and PDI, respectively, were detected using the anti-KDEL specific antibody, but were not detected in the secretion medium.

Several studies have shown that mature virus particles associate or contain some host cytoplasmic proteins during transport along the secretory pathway en route from the replication complexes to the plasma membrane [12]. We have shown that two positive-sense RNA viruses, the JEV and enterovirus 71 (EV71), induced the release of host intracellular proteins from virus-infected cells [9,10,13]. The virus-induced host cytoskeleton proteins and molecular chaperones, including the Hsp90β, Hsc70 and GRP78, are two major groups of secreted virus-associated cellular proteins that were characterized from the DENV, JEV and EV71, as reported by Higa *et al.* [14] and our previous studies [9,10,13]. GRP78, an ER-lumen protein, was also reported as present in different subcellular localization, including the cell plasma membrane, cytoplasm, mitochondria, nucleus and even the extracellular secretions of tumor cells. During virus infection, GRP78 can facilitate the proper folding and assembly of viral particles and, thus, promotes viral maturation and subsequent cellular infections. In addition, virus infection induces an ER stress response and the expression of some ER lumen proteins. We examined the induced expression of ER lumen proteins, including GRP94, GRP78 and PDI, as well as the detection of extracellular ER lumen proteins upon JEV infection. As shown in Figure 1, the levels of induced expression of GRP94, GRP78 and PDI were detected in Western blotting analyses by using specific antibodies. Protein bands of interest were also detected in the cultured medium from JEV-infected cells, but not in that from mock-infected cells, indicating that the secretion of the ER lumen proteins was virus induced. Notably, β-actin, a cytoskeleton protein, was not detected in the cultured medium from JEV-infected cells, indicating that the secretion of ER lumen proteins is not a result of cell lysis. However, we detected no protein band in the cultured medium from JEV-infected cells when the anti-KDEL specific antibody was employed for the same Western blotting analysis (Figure 1, Panel 2). We noticed that the cleavage of KDEL peptide was not easy to detect since this tetrapeptide is located at the C-terminus of a protein, therefore, it is impossible to determine a C-terminal peptide from a spot of interest on the polyacrylamide gel. However, as shown in Figure 1, we used the anti-KDEL antibody, which is a monoclonal antibody specific to KDEL tetrapeptide, exhibiting a high sensitivity of band intensities. By comparison with positive results, we showed that cleavage of KDEL is required for cytoplasmic GRP78 to participate in transportation of viral particles. Indeed, some reports have demonstrated that the cleavage of the carboxyl-terminal KDEL motif is critical for secretory transport of GRP78 toward the cell surface [15]; our results indicated that the cleavage of the KDEL, an ER-retention signal, is required for cytoplasmic GRP78 to participate in the folding, assembly and transport of viral particles during the virus infection cycle.

### 3.2. KDEL-Motif Is Not Essential for GRP78 Function in the Viral Protein Production

**Figure 2:**
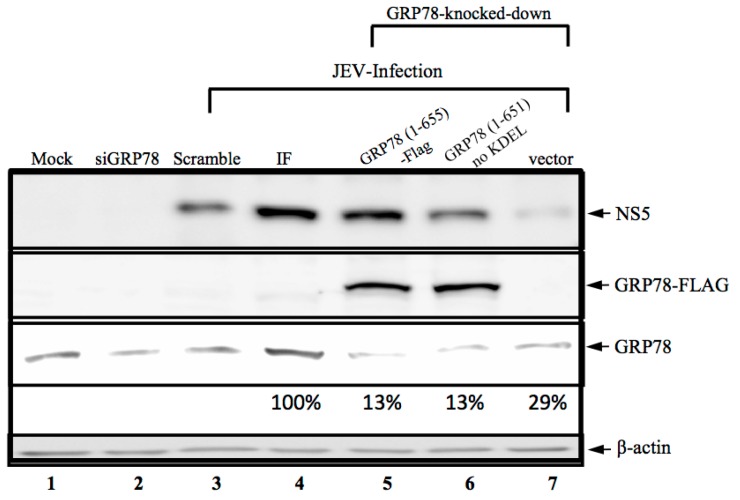
Ectopic expression of GRP78-Flag protein restores the JE viral protein production in the shGRP78 knocked down cells. BHK-21 cells were transfected with an antiGRP78 siRNA (Lane 2), scrambled siRNA (Lane 3) or infected with lentiviral vector containing the shGRP78-specific gene for generating GRP78 stable knocked down cells. The GRP78 knocked down cells were then overexpressed for full-length or KDEL-truncated GRP78 using transfected plasmids described in the Materials and Methods. Followed by infection with JEV, the cells were then harvested for the detection of viral nonstructural NS5 protein, GRP78-Flag tagged proteins, endogenous GRP78 and β-actin using the specific antibodies as indicated at the right. For the expression levels of GPR78 in wild-type and GRP78 stable knocked down cells, the quantitation of values of each lane are shown below the lane in the blot. Lanes assignments: Lane 1, BHK-21 cells used for negative control; Lane 2, the siGRP78 transfected cells; Lane 3, the scrambles siRNA transfected BHK-21 cells and infection with JEV; Lane 4, BHK-21 cells infected with JEV; Lane 5, the ectopically-expressed GRP78 protein (GRP78 (1-655)-Flag) and infection with JEV in the GRP78 knocked down cells; Lane 6, the ectopically-expressed KDEL-truncated GRP78 protein (GRP78 (1-651)-Flag) and infection with JEV in the GRP78 knocked down cells; Lane 7, the ectopically expressed Flag and infection with JEV in the GRP78 knocked down cells.

GRP78 has long been recognized as the ER stress protein and as a facilitator of viral replication. We demonstrated that the virus-associated GRP78, in its KDEL-motif free form (Figure 1), subsequently affected cellular infections. We considered whether this KDEL-motif within GRP78 was involved in viral replication. To address this, we created a shGRP78-stable knockdown of BHK-21 cells by transfecting them with the plasmid containing the shGRP78-specific targeted sequence and selecting for stable cells, in which were expressed extremely low amounts of GRP78. The overexpression of ectopic full-length GRP78-Flag (amino acid residues 1–655) or KDEL-truncated (amino acid Residues 1 to 651, no KDEL) GRP78 was then analyzed for shGRP78-stable cells to investigate their effect on JE viral replication. As expected, the knocking down of GRP78 resulted in the significantly reduced production of viral NS5 protein (Figure 2, Lane 2), indicating that GRP78 is critical for viral protein production. Next, overexpression of full-length or KDEL-truncated GRP78 protein restored the production of viral NS5 protein (Figure 2, Lanes 5 and 6), implying that the GRP78 function in viral infection cycle, especially the viral protein production, occurs in a KDEL-motif-independent manner.

### 3.3. KDEL-Motif Is Required for GRP78 Function in the Release of JE Viral Virions

**Figure 3:**
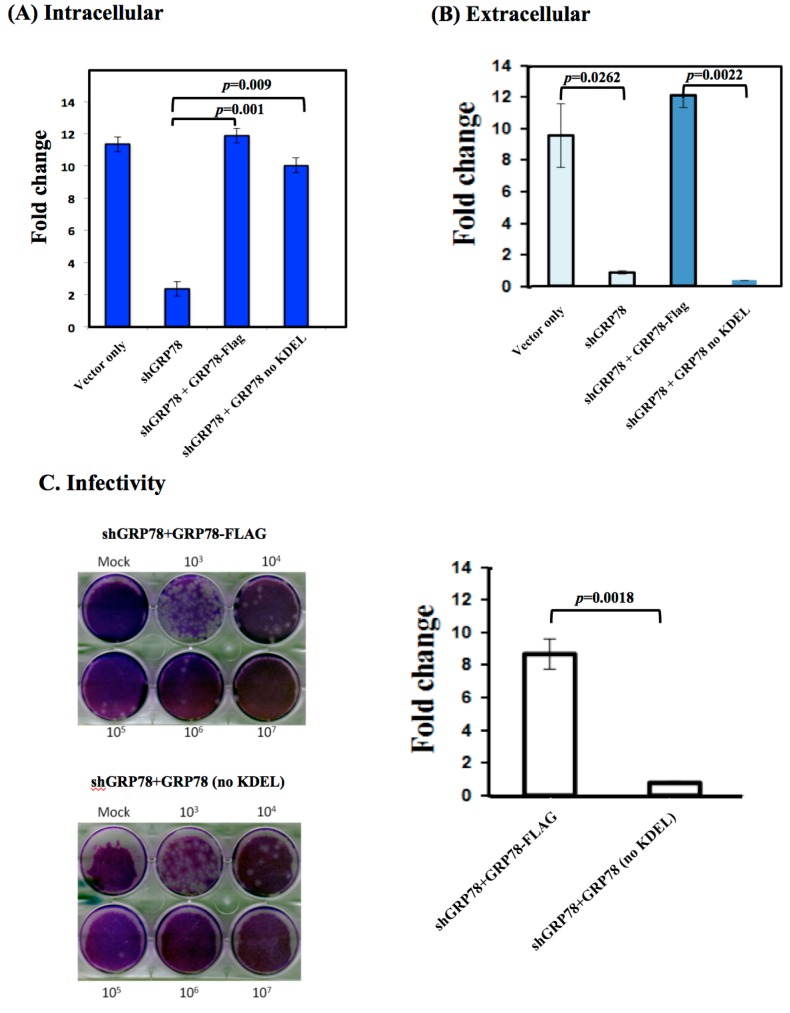
The C-terminal tetra-peptide KDEL within GRP78 is required for the production of infectious viral progeny. The GRP78 knocked down BHK-21 cells had ectopic overexpression of GRP78 or KDEL-motif-truncated GRP78 proteins followed by infection with JEV. (**A**) The cells were then harvested and prepared for the determination of JE viral RNA as described in the Materials and Methods; (**B**) The cultured medium was collected, and the JEV RNA was quantified by RT-qPCR as described in the Materials and Methods. The fold change of the JEV RNA level is defined as the relative increase amount of viral RNA in comparison to siGRP78-treated cells; (**C**) The viral progeny from the ectopically-expressed GRP78 or KDEL-truncated GRP78 proteins in the GRP78 knocked down cells were quantified by plaque assay. The virus titers are shown in PFU/mL. The data shown here are the means of three independent experiments with standard errors. The *p*-values indicate a significant difference between ectopically-expressed of GRP78 and KDEL-truncated GRP78 in the JEV-infected GRP78 knocked down BHK-21 cells. Abbreviations: vector only, BHK-21 cells were transfected with scramble siRNA followed by infection with JEV; shGRP78, the GRP78 stable knocked down BHK-21 cells infected with JEV; shGRP78 + GRP78-Flag, the GRP78 knocked down BHK-21 cells were ectopically expressed with GRP78-Flag protein, followed by infection with JEV; shGRP78 + GRP78-Flag no KDEL, the GRP78 knocked down BHK-21 cells were ectopically expressed with KDEL-motif-truncated GRP78 protein, followed by infection with JEV.

In BHK-21 cells, ER stress is a response to JEV infection and subsequently increases GRP78 expression in the ER compartment and promotes GRP78 relocalization from the ER to the cell surface. Munro and Pelham [16] reported that the C-terminal tetrapeptide KDEL motif prevents GRP78 trafficking from the ER to the plasma membrane. According to our previous studies, GPR78 is associated with viral enveloped proteins as viral particles move along the secretory pathway; therefore, we examined whether this KDEL motif is crucial for the release of a new viral progeny. In GRP78 knocked down cells, the overexpression of both full-length GRP78 and KDEL-truncated GRP78 restored the expression level of viral RNA with an amount similar to that of the viral RNA produced from the scrambled siRNA-treated cells (Figure 3A). However, the extracellular JE viral RNA levels were remarkably reduced in the overexpressed KDEL-truncated GRP78 cells compared to the overexpressed full-length GRP78 in the GRP78 knocked down and JEV-infected cells (Figure 3B). Consistently, no changes in viral infectivity were observed in the secreted virions from the overexpressed full-length GRP78 in the GRP78 knocked down and JEV-infected cells (Figure 3C, upper panel). By contrast, significantly reduced viral infectivity was observed in the secreted virions from overexpressed KDEL-truncated GRP78 in the GRP78 knocked down and JEV-infected cells (Figure 3C, lower panel). The quantified results for viral infectivity showed a minimum nine-fold reduction of secreted virions from the cells expressing GRP78 without the C-terminal KDEL protein (Figure 3D). These results suggested that this C-terminal KDEL-motif is required for the release of newly-formed viral progeny in the JEV infection cycle.

### 3.4. KDELR Protein Co-Fractionation with JE Viral Enveloped Proteins

**Figure 4:**
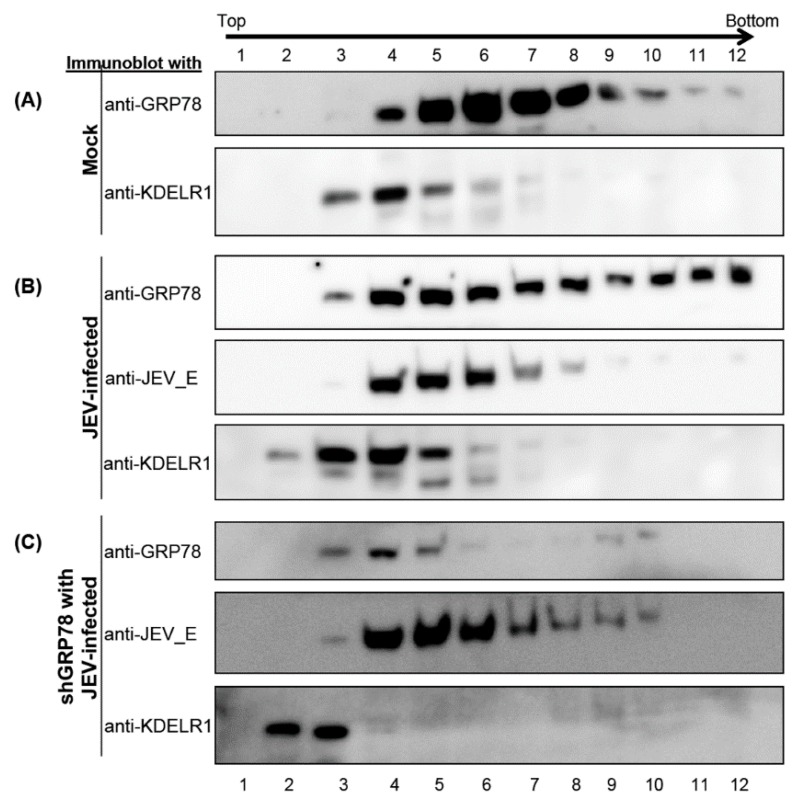
Co-purification of GRP78, KDELR and viral E protein from the JEV-infected cells. The BHK-21 cells were mock-infected or infected with JEV at the MOI of 0.1, and the cells were harvested by freeze-and-thaw for the disturbing of the cell membranes. The lysates were then subjected to a 30% sucrose cushion for the removal of nonspecifically interacting proteins from the virions. The JE viral particles were purified by isopycnic fractionation using a 20% to 60% linear sucrose density gradient. The JE viral E, GRP78 and KDEL receptor proteins were detected in the fraction by Western blotting using anti-JEV_E, anti-GRP78 and anti-KDELR1 antibodies, respectively. (**A**) Mock, BHK-21 cells with mock-infected; (**B**) JEV-infected, BHK-21 cells with JEV infection; (**C**) shGRP78 with JEV-infected, the GRP78 knocked down cells infected with JEV.

Many studies have suggested that the KDEL-retrieval system is involved in regulating the extent of GRP78 leaving the ER [17]. The KDELR protein plays a significant role in the folding of nascent polypeptides and secretory proteins in the KDEL-retrieval system [8]. In this study, we considered whether the KDELR protein was also involved in the maturation of viral particles, as we demonstrated that GRP78 binds to viral envelope proteins. Thus, we harvested the JEV-infected cell lysates and processed them by using a freeze-and-thaw treatment to disturb the cell membrane. The virions were then purified using a 20% to 60% sucrose density gradient. The copurification of GRP78 and the viral E and KDELR proteins was observed in two consecutive fractions (Figure 4B). Notably, the copurification of GRP78 and KDELR proteins was also present in the mock-infected control cells (A), implying the interaction of GRP78 and KDELR proteins in the normal cells. By contrast, purifying of the KDELR protein was found in various fractions from GRP78 and viral E proteins in the GRP78 knocked down and JEV-infected cells (Figure 4C). This result suggested that the KDELR protein is also co-fractionated with viral particles and that the co-fractionation between the KDELR protein and the virions is mediated by GRP78.

### 3.5. KDELR Protein is Engaged in the Subcellular Localization of JE Viral Particles in the Golgi

**Figure 5:**
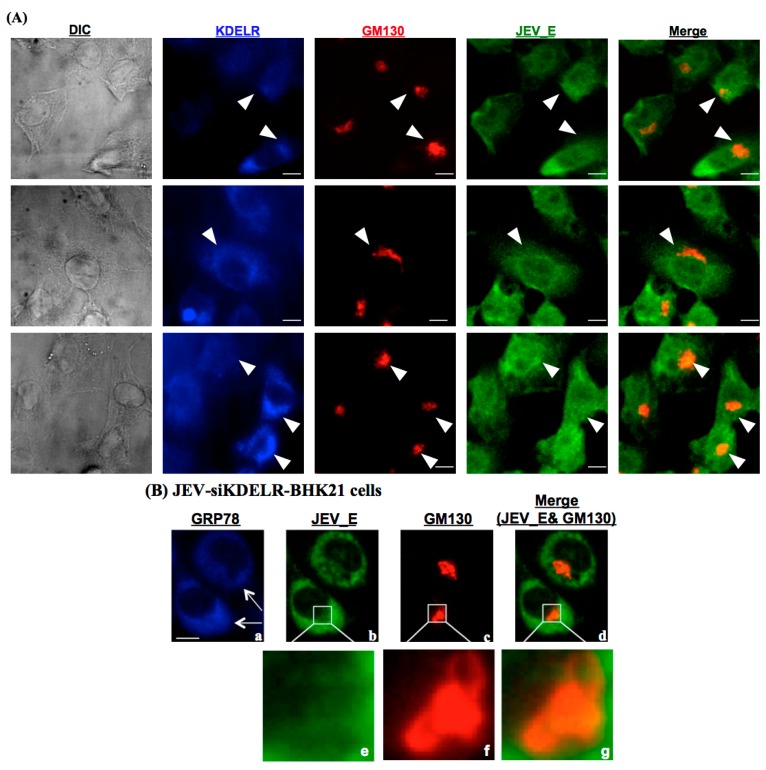
Interaction of KDELR protein and viral E protein was located in the Golgi membrane of JEV-infected cells. The BHK-21 cells were mock-infected or infected with JEV at an MOI of 0.1, followed by fixation and stained with antibodies in preparation for an immunofluorescence analysis that detected JEV viral E protein (green), GM130 (red) and KDELR protein (blue). (**A**) Subcellular localization of GRP78, GM130 and KDELR proteins in the JEV-infected cells. Differential interference contrast determined the cell morphology, as shown in the left panel. The positive stained signals were indicated as the white arrowhead. Bar = 10 μM; (**B**) The distinct subcellular localization of GRP78, JEV viral E protein and GM130 in the siKDELR-treated and JEV-infected BHK-21 cells. The anti-KDELR siRNA-transfected BHK-21 cells were infected with JEV and then harvested for immunofluorescence analyses with antibodies, as indicated. The positive stained of JEV viral E protein was indicated as the white arrow. Enlarged photos of (**b,c,d**) are shown in the square areas (**e,f,g**).

Subsequently, the subcellular location for the association of the KDELR, GRP78 and JEV particles was investigated. After JEV infection, the ER is the critical intracellular site where viral replication and assembly occur; subsequently, the newly-folded viral particles are modified and exit from the ER. We performed a triple immunolocalization of JEV E, GRP78 and GM130 (a Golgi marker protein) in the JEV-infected BHK-21 cells (Figure 5). At 48 h post-infection, the viral E protein was predominately localized at GM130; the Golgi membrane protein and KDELR protein were also colocalized with both proteins, indicating that the JEV particles were trafficked from the ER to the Golgi (Figure 5A). We then knocked down the expression of KDELR protein through transfection with specific siRNA; the viral E protein was not localized at GM130, the Golgi membrane protein (Figure 5B). We also observed that no changes occurred in the expression of GRP78 in the siKDELR cells, indicating that GRP78 and the KDELR proteins were involved in the JEV infection cycle as a sequential event.

### 3.6. KDELR Protein Interacts with Viral Structural Proteins, but Not with Nonstructural NS1 Protein

**Figure 6:**
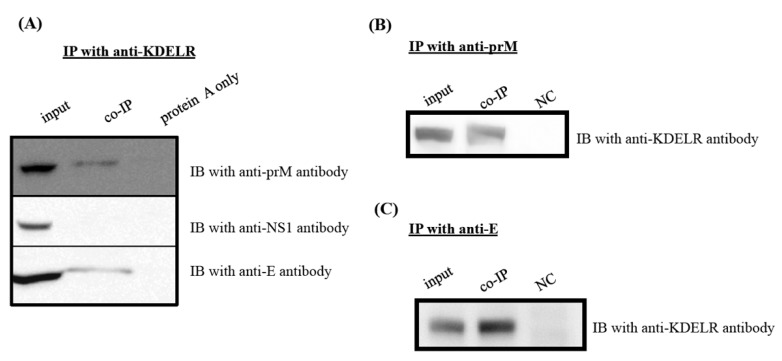
KDELR protein binds to JE viral structural proteins, but not NS1 protein. Cell lysates of BHK-21 cells with mock-infected or JEV-infected for 48 h were immunoprecipitated (IP) with anti-KDELR antibody (**A**), anti-prM antibody (**B**) and anti-E antibody (**C**), respectively. The membranes were then analyzed with various specific antibodies as indicated; (**A**) The IP membranes were immunoblotting with anti-prM-, anti-NS1- and anti-E-specific antibodies, respectively; (**B,C**) the IP membrane was immunoblotted with anti-KDELR antibody. Please note that cell lysates alone are shown on the left side as input reference. IB, immunoblot; IP, immunoprecipitation.

We examined whether KDELR protein interacted with the structural or nonstructural proteins of the JEV particles by using immunoprecipitation (Figure 6). As expected, the intracellular interaction between the KDELR protein and the viral prM and E proteins was confirmed using co-immunoprecipitation with anti-KDELR specific antibodies (Figure 6A). However, no interaction was found between the NS1 and viral nonstructural proteins from the eluates of the co-immunoprecipitation with anti-KDELR antibody reactions. Similarly, the interaction between KDELR and viral structural proteins was further confirmed by co-immunoprecipitation with either anti-prM (Figure 6B) or anti-E (Figure 6C) antibodies. These results indicated that the KDELR protein participated in the trafficking of JEV particles along the ER-Golgi pathway.

### 3.7. Impaired Secretory Trafficking of JEV Particles in the KDELR Knocked down Cells

**Figure 7:**
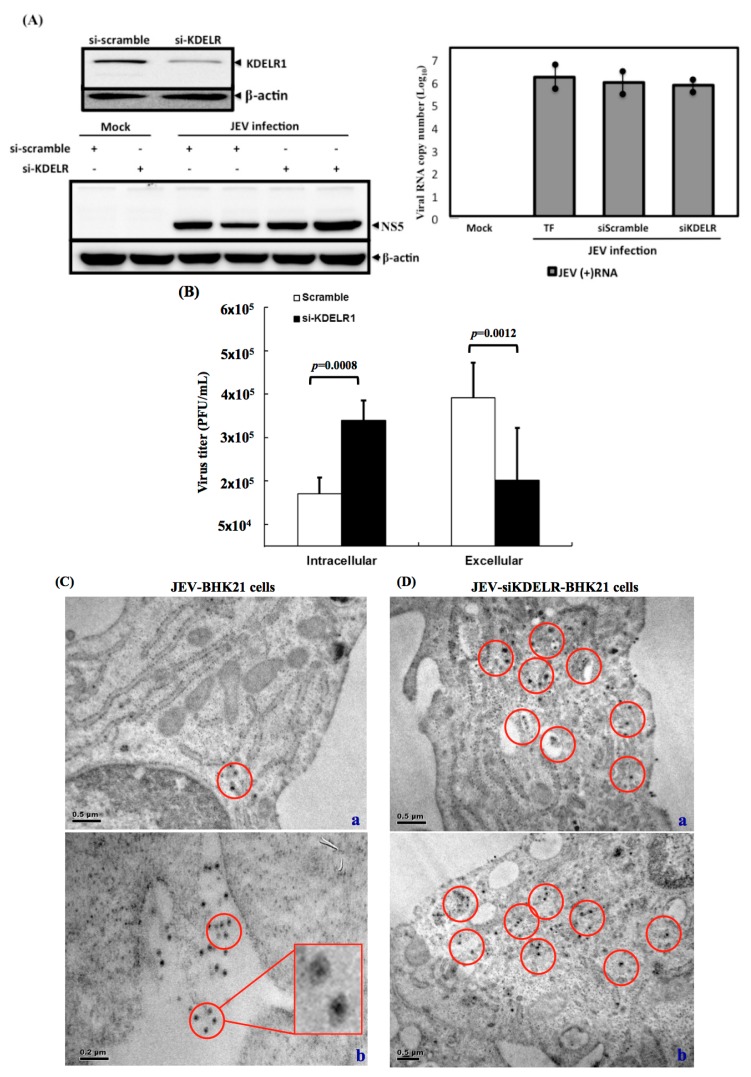
Knockdown of KDELR protein resulted in the reduction of extracellular viral infectivity in the BHK-21 cells. (**A**) Knockdown of KDELR protein does not affect JE viral replication. The reduced expression of KDELR protein was indicated in the siKDELR-treated cells. The production of viral protein, NS5 and accumulation of viral RNA were not affected in the anti-KDELR siRNA transfected compared to the anti-scramble siRNA transfected BHK-21 cells (TF: transfection reagent only); (**B**) The intracellular accumulated JE viral particles were detected in the siKDELR-treated cells. The anti-KDELR siRNA transfected or anti-scramble siRNA transfected BHK-21 cells were infected with JEV for 48 h. The JE virions were collected from cell lysate processing with freeze-and-thaw (intracellular) or the cultured medium (extracellular). The infectivity of JEV progeny from either intracellular or extracellular samples was performed in the plaque assay. The virus titers are shown in PFU/mL. Three independent knockdown experiments were performed; (**C,D**) Ultrastructural analysis of the JEV-infected BHK-21 cells; (**C**) The si-scramble-treated transfected BHK-21 cells were infected with JEV and harvested at 48 h for ultrastructural analysis using electron microscope; JEV electron dense particles (showed in red circle) can be observed in close proximity with the plasma membrane (**a**); in particular, many JEV electron dense particles were found outside of the plasma membrane (**b**). An enlarged photo was inserted to indicate that dense particles were JE virions; (**D**) In the siKDELR-treated cells, the JEV electron dense particles (shown in the red circles) were found in close proximity to the replication complexes (**a,b**). An representative enlarged photo of JEV electron dense particles was shown in the red box. No similar structures were seen outside of the plasma membrane in the siKDELR transfected cells.

To understand the effect of the KDELR on the JEV particles, we then determined the effect of reducing KDELR expression on JEV particle release. Three experiments were conducted to explore the functional events of KDELR involvement in the intracellular trafficking of viral particles. Firstly, knockdown of KDLER protein did not affect the JE viral RNA replication and viral protein production in the JEV-infected BHK-21 cells (Figure 7A). Secondly, the viral infectivity of the intracellular-assembled virions or extracellular-released virions was determined using a plaque-forming assay. As shown in Figure 7B, the significantly higher viral infectivity measured in extracellular virions was comparable with that in intracellular virions, suggesting that the assembly virions are released outside the plasma membrane en route along the ER-Golgi secretory pathway. By contrast, the accumulated high viral infectivity of the intracellular virions was observed in the KDELR-specific siRNA-treated cells (Figure 7B), implying that the KDELR protein played a role in viral particle transport along the secretory pathway in JEV-infected BHK-21 cells. Finally, ultrastructural analysis, performed using a transmission electron microscope, was employed to investigate the cellular distribution of JEV particles. As expected, only a few virus particles were observed in the wild-type JEV-infected BHK-21 cells, although virus particles were seen outside the cells near the plasma membrane (Figure 7C). By contrast, an accumulation of virus particles was observed in close association with the ER and replication complexes of the siKDELR-treated cells (Figure 7D). These results strongly suggested that the KDELR protein, an ER-Golgi retrieval system component, mediated the intracellular trafficking of JEV particles. Thus, in this study, we demonstrated the export of JEV particles to be mediated by the host ER-Golgi retrieval system components along the secretory pathway.

## 4. Discussion

We identified the KDELR protein, an ER-Golgi retrieval system component, as a host of JE viral particles in intracellular trafficking. GRP78, traditionally well recognized as a major ER chaperone, facilitates the trafficking of assembled viral particles by interacting with viral structural proteins. In contrast to some host and viral proteins, whose expression can be deleted to determine whether their roles are required, such an undertaking was unfeasible for either the KDELR protein or GRP78, because they are essential for maintaining cell homeostasis. In this study, we either knocked down the expression of GRP78 to demonstrate the involvement of the KDELR protein in virion preparation (Figure 4C) or determined the accumulation of intracellular (but not extracellular) high viral infectivity in KDELR-specific siRNA-treated cells (Figure 7). Thus, we carefully reduced KDELR proteins and GRP78 in minimal ways to assess whether both of the proteins were crucial in the intracellular trafficking of viral particles.

In JEV, the mechanism for trafficking newly-assembled viral particles from the ER to the plasma membrane remains unknown. In this study, the assembled viral particles, generated by translating viral envelope proteins produced at the ER-derived membranes, were associated with GRP78, an ER luminal protein, and led to a connection with the ER-Golgi retrieval system by interacting with a KDELR protein through its C-terminal tetrapeptide KDEL motif. We knew that the KDELR protein is typically transported between the ER and Golgi [18]. Therefore, in viral-infected cells, the virus benefits from host transport pathways that operate between the two host compartments, presumably to assist their newly-assembled viral particles in exiting from the ER. Recently, Li *et al.* [19] reported that dengue virus prM protein interacted with the KDEL receptor in the ER. They further demonstrated that blocking the host KDELR retrieval system led to reducing the viral particles’ release, indicating that KDELR protein is an intracellular viral receptor assisting in DENV exit from the ER [20]. We noticed that enteroviral infections could reorganize the cellular secretory pathway, including the generation of PI4P lipid-enriched replication organelles; the PI4P lipid-rich membrane led to facilitated viral RNA replication, instead of using the conventional secretory pathway for the intracellular trafficking of viral particles [6]. Instead, we found that JE viral structural proteins bound to GRP78, an ER chaperone, through a KDELR-related mechanism; the virus likely recruited the host ER retrieval system for assisting assembled viral particles in exiting from the ER and translocating to the Golgi complex for their subsequent exit from the cell.

JE virus depends entirely on the host proteins to perform numerous functions during the infection replication. Virus infection often leads to hijacking many host cellular factors and machineries for completing its life cycle. The intracellular trafficking of progeny virus using the intracellular membrane transport involves the well-coordinated engagement of some organelles that ensure that the viral particles are delivered to their correct destination, the Golgi complex, for the secretion of JE viral particles. We and other groups have reported that GRP78 and calreticulin interact with misfolded aggregates containing HCV/JEV viral proteins and are likely to be involved in their repair. GRP78 exhibits the retrieval capacity through the C-terminal tetrapeptide, KDEL. At the ER, some proteins are synthesized co-translationally and then folded by chaperones and exported to the Golgi complex. The KDELR proteins are transmembrane proteins cycling between ER and Golgi apparatus to ensure the corrected folding of ER-resident protein, such as GRP78, and retrieve them back to the ER. In this study, we demonstrated that, upon JEV infection, virus will then make use of the host KDELR retrieval system for the translocation of progeny virus from budding sites in the ER to the Golgi.

Munro and Pelham revealed the mechanisms for GRP78 trafficking from the ER to the plasma membrane [16]. A featured tetrapeptide KDEL motif maintains GRP78 within the ER lumen. The upregulated expression in the intracellular level of GRP78 triggered by ER stress from virus infections has been well documented [9,21]. By contrast, a corresponding upregulation of KDELR protein expression did not occur under ER stress in our study (data not shown). An excess expression of GPR78 may be associated with viral structural proteins to form particles termed “GRP78-associated assembled viral particles”. Within a relatively short period, the overproduction of GRP78-associated assembled viral particles may exceed the retention capacity of the KDEL retrieval system, resulting in an escape from the ER to the Golgi and a subsequent exiting to the cell surface. Another possible mechanism for the GRP78-associated transport of assembled JEV particles from the ER to the Golgi is the activity of various components of the KDEL system altered on viral infection and resulting in facilitating the GRP78 intracellular trafficking and subsequent exit from the ER. Additionally, GRP78 transport may involve a masking of the KDEL motif by glycosylation or other protein modifications to the sequence adjacent to the C-terminal of the KDEL tetrapeptide. Zhang *et al.* [15] reported that some glycosylation sites existed at the C-terminus of GRP78 and that these sites were in proximity to the KDEL motif [15,22]. However, in our study, we detected no glycosylated form of GRP78 in the JEV-infected BHK21 cells. Future study is necessary to fully determine the GRP78 characteristics involved in the intracellular trafficking of viral particles.

## 5. Conclusions

In conclusions, we show that the KDELR protein is a critical host factor for assisting newly-formed viral particles to exit from the ER. The association between the KDELR protein and viral particles is mediated by GRP78, an ER lumen protein. When the BHK21 cells are infected with JEV, the intracellular export of JEV particles is mediated by the host ER-Golgi retrieval system components along the secretory pathway.
